# Perioperative Blood Transfusion as a Significant Predictor of Biochemical Recurrence and Survival after Radical Prostatectomy in Patients with Prostate Cancer

**DOI:** 10.1371/journal.pone.0154918

**Published:** 2016-05-09

**Authors:** Jung Kwon Kim, Hyung Suk Kim, Juhyun Park, Chang Wook Jeong, Ja Hyeon Ku, Hyun Hoe Kim, Cheol Kwak

**Affiliations:** Department of Urology, Seoul National University Hospital, Seoul, Korea; University of Kentucky, UNITED STATES

## Abstract

**Purpose:**

There have been conflicting reports regarding the association of perioperative blood transfusion (PBT) with oncologic outcomes including recurrence rates and survival outcomes in prostate cancer. We aimed to evaluate whether perioperative blood transfusion (PBT) affects biochemical recurrence-free survival (BRFS), cancer-specific survival (CSS), and overall survival (OS) following radical prostatectomy (RP) for patients with prostate cancer.

**Materials and Methods:**

A total of 2,713 patients who underwent RP for clinically localized prostate cancer between 1993 and 2014 were retrospectively analyzed. We performed a comparative analysis based on receipt of transfusion (PBT group vs. no-PBT group) and transfusion type (autologous PBT vs. allogeneic PBT). Univariate and multivariate Cox-proportional hazard regression analysis were performed to evaluate variables associated with BRFS, CSS, and OS. The Kaplan-Meier method was used to calculate survival estimates for BRFS, CSS, and OS, and log-rank test was used to conduct comparisons between the groups.

**Results:**

The number of patients who received PBT was 440 (16.5%). Among these patients, 350 (79.5%) received allogeneic transfusion and the other 90 (20.5%) received autologous transfusion. In a multivariate analysis, allogeneic PBT was found to be statistically significant predictors of BRFS, CSS, and OS; conversely, autologous PBT was not. The Kaplan-Meier survival analysis showed significantly decreased 5-year BRFS (79.2% vs. 70.1%, log-rank, p = 0.001), CSS (98.5% vs. 96.7%, log-rank, p = 0.012), and OS (95.5% vs. 90.6%, log-rank, p < 0.001) in the allogeneic PBT group compared to the no-allogeneic PBT group. In the autologous PBT group, however, none of these were statistically significant compared to the no-autologous PBT group.

**Conclusions:**

We found that allogeneic PBT was significantly associated with decreased BRFS, CSS, and OS. This provides further support for the immunomodulation hypothesis for allogeneic PBT.

## Introduction

Transfusion-related immunomodulation (TRIM), including alloimmunization, tolerance, and immunosuppression [1], has been postulated to explain the association between perioperative blood transfusion (PBT) and survival in a number of malignancies, including colon, esophageal, and hepatic carcinomas [2–4]. However, it is difficult to conclude whether these oncologic outcomes are secondary to PBT itself or whether PBT serves as a surrogate marker for clinically important variables that may affect oncologic prognosis. Previous study suggested the several reasons–including obscuring the operative field, limiting anatomical visualization, and preventing full excision the tumor–to hypotheses for why an excessive blood loss followed by PBT might be correlated with the oncologic outcomes [5].

In urological cancers, there have been conflicting reports regarding the association of PBT with oncologic outcomes including recurrence rates and survival outcomes [6–14]. Specifically for radical prostatectomy (RP), to the best of our knowledge, there have been over 10 retrospective studies that examining the association between PBT and recurrence and/or survival after RP for prostate cancer [7–9, 15–23]. About one third of the studies suggested that PBT resulted in increased cancer recurrence and/or mortality [9, 20, 21, 23], while the others showed no significant associations [7, 8, 15–19, 22].

In the current study, we investigated whether PBT (autologous vs. allogeneic) affects biochemical recurrence-free survival (BRFS), cancer-specific survival (CSS) and overall survival (OS) after RP in patients with prostate cancer, by using a large tertiary referral center RP database.

## Materials and Methods

### Ethics Statement

The Institutional Review Boards (IRBs) of the Seoul National University Hospital approved this study (Approval number: H-1510-049-710). As the present study was carried out retrospectively, written informed consent from patients was waived by the IRBs. Personal identifiers were completely removed and the data were analyzed anonymously. Our study was conducted according to the ethical standards laid down in the 1964 Declaration of Helsinki and its later amendments.

### Study cohort

A total of 2,713 patients who underwent RP (open or laparoscopic or robot-assisted laparoscopic) for clinically localized prostate cancer between 1993 and 2014 at our institution were included in this study. Clinical data in the medical records were retrospectively reviewed. 46 cases were excluded because of insufficient clinical data; a total of 2,667 cases were investigated.

### Acquisition and definition of data

RPs were conducted by several surgeons during the involved period. All pathological specimens were evaluated by a staff pathologist who had genitourinary expertise. To perform a comparative analysis based on receipt of transfusion (PBT group vs. no-PBT group) and transfusion type (autologous PBT vs. allogeneic PBT), the following variables were included in current analysis: age, body mass index (BMI), D’Amico risk classification, preoperative hemoglobin (Hb), neoadjuvant androgen deprivation therapy (ADT), operative type (Open vs. laparoscopic vs. Robotic), conduction of pelvic lymph node dissection (PLND) and neurovascular bundle (NVB) saving, operative time, estimated blood loss (EBL), preoperative prostate specific antigen (PSA) level, pathologic tumor (pT) stage and Gleason score (pGS), lymph node (LN) status, total number of removed LN, number of positive LN, extracapsular extension (ECE), seminal vesicle invasion (SVI), surgical margin status (PSM), adjuvant ADT, adjuvant radiotherapy (RT), salvage RT, follow-up duration, biochemical recurrence (BCR) rate, CSS rate, and OS rate. The pathologic T stage was categorized as ≤ pT2 or ≥ pT3 (organ confined disease, or not), and pathologic GS was classified as GS ≤ 8 or GS > 8. Subgroup analysis was also performed in the patients who underwent open RP with EBL ≥ 1000ml to adjust for potential confounding factors.

According to our standardized postoperative protocol, we evaluated serum PSA level every 3 months for 1 year, then every 6 months for 4 additional years, followed by annually thereafter. BCR was defined as either two consecutive increasing PSA values of > 0.2 ng/mL or the conduction of adjuvant therapy during the postoperative follow-up period.

PBT was defined as transfusion of allogeneic or autologous red blood cells (RBCs) during RP or within the postoperative hospitalization. Transfusion of other blood products, including fresh frozen plasma or platelets, was not included in this analysis. The administration of PBT was based on the volition of the physicians. No institutional intraoperative or postoperative standardized criteria were used for transfusion.

### Statistical analyses

The clinicopathological characteristics were compared between PBT group and no-PBT group using chi-squared test for categorical variables, and independent t-test or Mann-Whitney U test for continuous variables. The Kaplan-Meier method was used to calculate survival estimates for BRFS, CSS, and OS, and log-rank test was used to conduct comparisons between the groups. Univariate and multivariate Cox-proportional hazard regression analysis were performed to evaluate significant variables associated with BRFS, CSS, and OS. The following factors were included in the analysis: age, BMI, D’Amico risk classification, preoperative Hb, neoadjuvant ADT, operative type, conduction of PLND and NVB saving, operative time, EBL, preoperative PSA, pathologic T stage and GS, LN status, total number of removed LN, number of positive LN, ECE, SVI, PSM, adjuvant ADT and RT, salvage RT, follow-up duration, PBT, allogeneic PBT, and autologous PBT. All statistical analyses were performed using commercially available software (IBM SPSS Statistics ver. 21.0, Armonk, NY, USA) and two-sided p-values of <0.05 were considered statistically significant.

## Results

Mean patient age was 66.2 ± 6.9 years, and the median follow-up period was 60.2 (range 0–261) months. The number of patients who received PBT was 440 (16.5%). Among these patients, 350 (79.5%) received allogeneic with or without autologous transfusion (allogeneic PBT group), and the other 90 (20.5%) received only autologous transfusion (autologous PBT group). In the comparative analysis of clinicopathological features between the PBT group and no-PBT group, patients in the PBT group showed a higher rate of high -risk patients according to D’Amico risk classification, a lower preoperative Hb level, a higher rate of open RP (ORP) compared to laparoscopic (LRP) or robotic RP (RARP), a higher frequency of PLND, a lower frequency of NVB saving, a longer operative time, a higher EBL, higher pathologic GS, a larger number of removed LNs, a higher frequency of salvage RT, longer follow-up duration and higher rates of BCR, CSS, and OS in comparison with the no-PBT group (Table 1). In subgroup analysis comparing the allogeneic and autologous PBT group, patients in allogeneic PBT group showed younger age (p = 0.001), a higher rate of LRP/RARP compared to ORP (p = 0.007), a high frequency of PLND (p < 0.001), a longer operative time (p < 0.001), a higher EBL (p < 0.001), a higher frequency of salvage RT (p < 0.001), higher rates of BCR (p < 0.001), cancer-specific death (p = 0.014), and all-cause death (p = 0.001). The other pathologic variables, however, were not significantly different between the two groups (S1 Table). In addition, no significant differences were observed based on categorization of the number of transfused units (1 unit vs. 2 units vs. more than 2 units, data not shown).

**Table 1:** Clinicopathological parameters of the comparative analysis results according to the presence or absence of perioperative blood transfusion (PBT).

	PBT	No-PBT	p-value
Patient n (%) = 2667	440 (16.5%)	2227 (83.5%)	
Allogeneic PBT*, n (%)	350 (79.5%)		
Autologous PBT, n (%)	90 (20.5%)		
Age, yr, median (SD)	66.2 (7.4)	66.3 (6.7)	0.752
BMI, median (SD)	24.2 (2.7)	24.3 (2.7)	0.667
D’Amico classification^**+**^, n (%)			0.024
Low—risk	128 (13.8%)	798 (86.2%)	
Intermediate—risk	188 (17.7%)	875 (82.3%)	
High—risk	124 (18.3%)	554 (81.7%)	
Preoperative Hb, g/d L, mean (SD)	13.8 (1.4)	14.2 (1.2)	<0.001
Neoadjuvant ADT, n (%)			0.477
Done	19 (4.3%)	116 (5.2%)	
Not done	421 (95.7%)	2111 (94.8%)	
Operative type, n (%)			<0.001
Open	380 (86.3%)	1283 (57.6%)	
Laparoscopic	13 (3.0%)	46 (2.1%)	
Robotic	47 (10.7%)	898 (40.3%)	
PLND, n (%)			<0.001
Done	164 (38.3%)	427 (20.4%)	
Not done	264 (61.7%)	1666 (79.6%)	
NVB saving, n (%)			0.003
Done	66 (21.7%)	383 (30.2%)	
Not done	238 (78.3%)	887 (69.8%)	
Operative time, min, median (SD)	188.1 (82.1)	173.2 (110.3)	0.007
EBL, ml, median (SD)	1165.8 (1007.3)	591.1 (411.1)	<0.001
Preoperative PSA, ng/ml, mean (SD)	13.5 (17.8)	12.6 (18.9)	0.368
Pathologic Gleason Score, n (%)			0.001
≤ 8	353 (84.4%)	1897 (90.1%)	
>8	65 (15.6%)	208 (9.9%)	
Pathologic T stage, n (%)			0.889
≤ pT2	266 (60.5%)	1336 (60.1%)	
≥pT3	174 (39.5%)	887 (39.9%)	
Lymph node status, n (%)			0.546
Nx/N0	422 (95.9%)	2148 (96.5%)	
N1	18 (4.1%)	78 (3.5%)	
Total number of removed lymph nodes, mean (SD)	3.8 (5.8)	3.1 (4.0)	0.011
Number of positive lymph nodes, mean (SD)	0.08 (0.5)	0.09 (0.7)	0.737
ECE, n (%)			0.477
Absent	286 (65.0%)	1404 (63.2%)	
Present	154 (35.0%)	817 (36.8%)	
SVI, n (%)			0.159
Absent	375 (85.2%)	1953 (87.7%)	
Present	65 (14.8%)	274 (12.3%)	
PSM, n (%)			0.499
Absent	278 (63.2%)	1442 (64.9%)	
Present	162 (36.8%)	781 (35.1%)	
Adjuvant ADT, n (%)			0.324
Done	15 (3.4%)	55 (2.5%)	
Not done	425 (96.6%)	2172 (97.5%)	
Adjuvant radiotherapy, n (%)			0.118
Done	11 (2.4%)	27 (1.2%)	
Not done	429 (97.6%)	2200 (98.8%)	
Salvage radiotherapy, n (%)			<0.001
Done	71 (16.1%)	183 (8.2%)	
Not done	361 (83.9%)	2044 (91.8%)	
Follow-up, months, median (SD)	79.4 (54.2)	56.5 (34.4)	<0.001
Biochemical recurrence, n (%)			<0.001
No	338 (76.8%)	1882 (84.7%)	
Yes	102 (23.2%)	341 (15.3%)	
CSS result, n (%)			<0.001
Alive or death from other causes	422 (95.9%)	2200 (98.8%)	
Cancer-specific death	18 (4.1%)	27 (1.2%)	
OS result, n (%)			<0.001
Alive	388 (88.2%)	2129 (95.6%)	
All-cause death	52 (11.8%)	98 (4.4%)	

* allogeneic with/without autologous PBT

^**+**^ Low-risk: PSA ≤ 10, Gleason score ≤ 6, and clinical stage T1-2a, Intermediate-risk: 10 < PSA < 20, Gleason score 7, or clinical stage T2b, High-risk: PSA ≥ 20, Gleason score ≥ 8, or clinical stage T2c-3a

ADT: androgen deprivation therapy, BMI: body mass index, CSS: cancer-specific survival, EBL: estimated blood loss, ECE: extracapsular extension, Hb: hemoglobin, NVB: neurovascular bundle, OS: overall survival, PLND: pelvic lymph node dissection, PSM: positive surgical margin, PBT: perioperative blood transfusion, SVI: seminal vesical invasion.

### Oncologic outcomes

**Fig 1:**
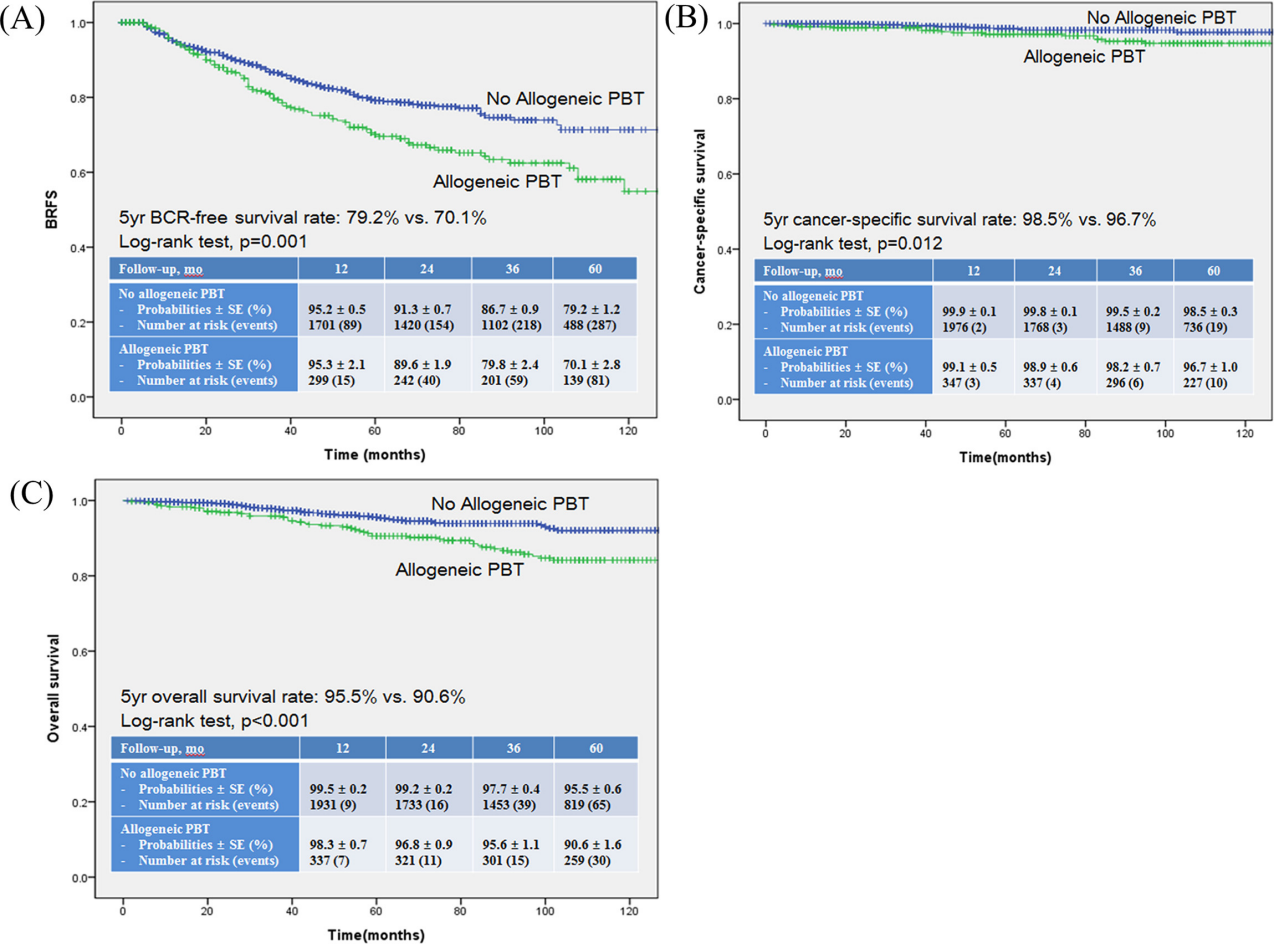
**Kaplan-Meier survival curves** for (A) BCR-free survival (BRFS), (B) Cancer-specific survival (CSS) and (C) Overall survival (OS) according to the administration of allogeneic perioperative blood transfusion (PBT).

**Fig 2:**
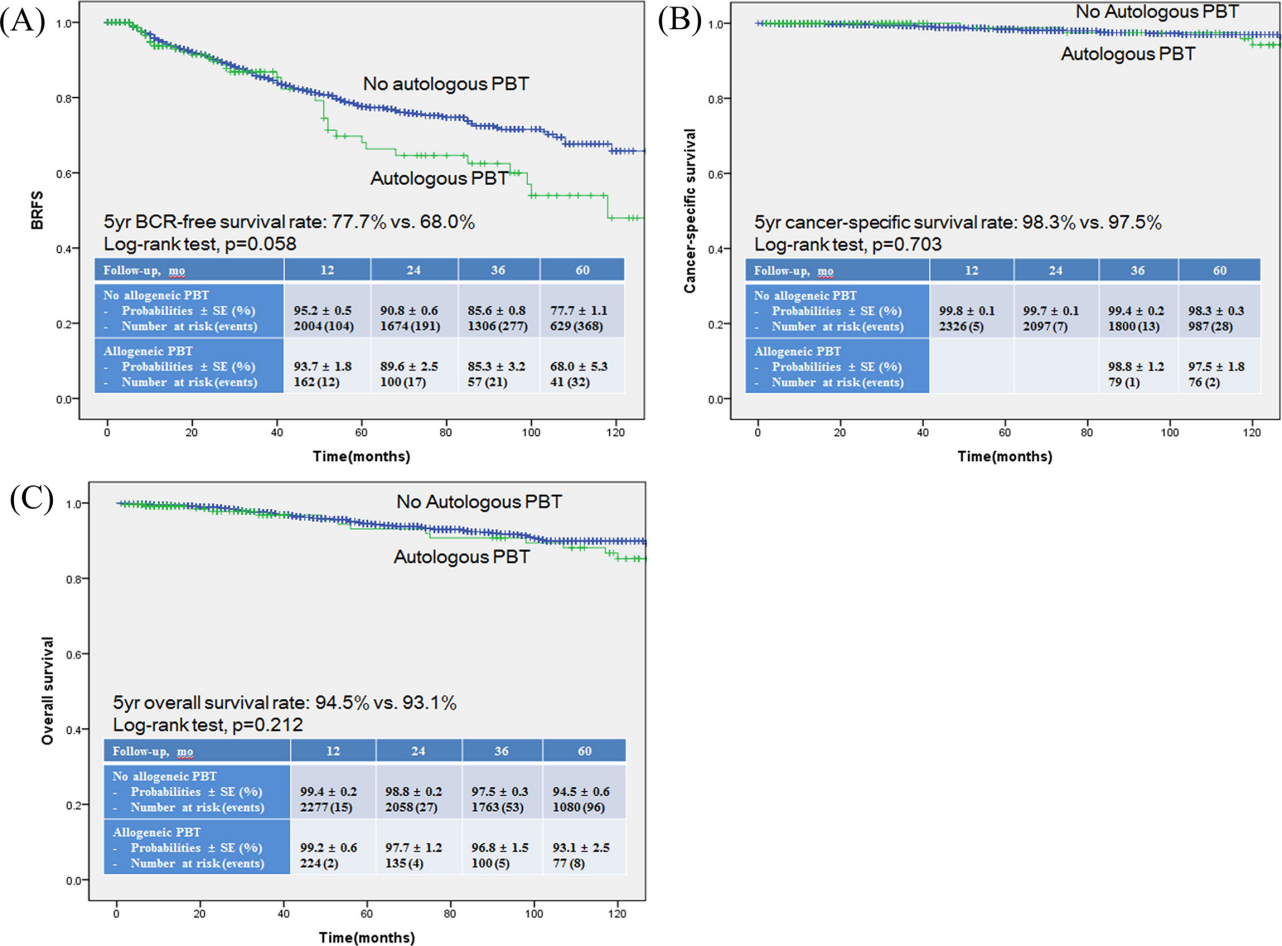
**Kaplan-Meier survival curves** for (A) BCR-free survival (BRFS), (B) Cancer-specific survival (CSS) and (C) Overall survival (OS) according to the administration of autologous perioperative blood transfusion (PBT).

On using a multivariate Cox regression analysis, D’Amico risk classification, pathologic T stage, pathologic GS, PSM, and allogeneic PBT were found to be statistically significant predictors of BRFS (Table 2). Additionally, age, D’Amico risk classification, pathologic GS, SVI, follow-up duration, and allogeneic PBT were identified as significant predictors of CSS (Table 2), while age, D’Amico risk classification, pathologic GS, SVI, follow-up duration, PBT, and allogeneic PBT were found to be significant predictors of OS (Table 2). Conversely, autologous PBT was not identified as significant predictor in either univariate or multivariate analysis for BRFS, CSS, and OS. The Kaplan-Meier survival analysis showed significantly decreased BRFS, CSS, and OS in the allogeneic PBT group compared to the no-allogeneic PBT group (Fig 1). In the autologous PBT group, however, none of these were statistically significant compared to the no-autologous PBT group (Fig 2).

**Table 2:** Multivariate Cox regression analyses for evaluating variables associated with BCR-free survival (BRFS), cancer-specific survival (CSS), and overall survival (OS).

	BRFS		CSS		OS	
	HR (95% CI)	p-value	HR (95% CI)	p-value	HR (95% CI)	p-value
Age	1.004	0.590	1.088	0.037	1.064	0.001
	(0.988–1.021)		(1.005–1.177)		(1.025–1.104)	
BMI	0.989	0.577	0.902	0.316	0.929	0.089
	(0.950–1.029)		(0.737–1.104)		(0.853–1.011)	
D’Amico classification^**+**^, n (%)						
Low—risk	Reference		Reference		Reference	
Intermediate—risk	2.036	<0.001	4.030	0.001	1.535	0.046
	(1.573–2.637)		(2.615–16.516)		(1.007–2.340)	
High—risk	2.430	<0.001	6.713	0.001	2.226	<0.001
	(1.857–3.180)		(2.704–25.734)		(1.449–3.421)	
Preoperative Hb	1.005	0.150	0.669	0.209	0.725	0.146
	(0.998–1.012)		(0.357–1.253)		(0.470–1.119)	
Neoadjuvant ADT	1.711	0.365	2.270	0.465	1.890	0.579
	(0.536–5.467)		(0.534–4.656)		(0.199–17.916)	
Operative type						
Open	Reference		Reference		Reference	
Laparoscopic/Robotic	0.788	0.102	1.888	0.320	0.662	0.182
	(0.592–1.049)		(0.539–6.605)		(0.362–1.213)	
PLND	1.282	0.095	1.024	0.972	0.555	0.114
	(0.957–1.717)		(0.265–3.960)		(0.268–1.151)	
NVB saving	0.858	0.336	1.070	0.918	1.301	0.447
	(0.629–1.172)		(0.297–3.856)		(0.660–2.564)	
Operative time	1.000	0.506	1.001	0.166	1.001	0.555
	(0.999–1.001)		(0.999–1.003)		(1.000–1.002)	
EBL	1.000	0.971	1.000	<0.001	1.000	0.007
	(1.000–1.000)		(1.000–1.001)		(1.000–1.000)	
Preoperative PSA	1.005	0.150	1.091	0.023	1.001	0.807
	(0.998–1.012)		(1.006–1.180)		(0.991–1.011)	
Pathologic T stage						
≤pT2	Reference		Reference		Reference	
≥pT3	2.517	0.001	1.039	0.976	1.315	0.606
	(1.488–4.257)		(0.080–13.519)		(0.464–3.725)	
Pathologic GS						
≤ 8	Reference		Reference		Reference	
>8	1.625	0.001	6.967	<0.001	1.507	0.042
	(1.216–2.171)		(2.590–18.744)		(1.014–2.246)	
LN status						
Nx/N0	Reference		Reference		Reference	
N1	0.608	0.176	4.183	0.099	1.148	0.760
	(0.296–1.250)		(0.766–22.847)		(0.474–2.781)	
Total number of removed LN	1.011	0.344	0.872	0.055	0.964	0.222
	(0.988–1.035)		(0.759–1.003)		(0.909–1.022)	
Number of positive LN	1.100	0.675	1.385	0.097	1.098	0.636
	(0.865–1.400)		(0.943–2.035)		(0.745–1.619)	
ECE	0.742	0.221	3.418	0.265	1.235	0.665
	(0.460–1.196)		(0.394–29.670)		(0.475–3.213)	
SVI	0.996	0.983	6.973	0.001	2.003	0.015
	(0.707–1.403)		(2.220–21.898)		(1.147–3.500)	
PSM	1.570	<0.001	1.096	0.883	1.384	0.150
	(1.239–1.990)		(0.325–3.698)		(0.890–2.153)	
Adjuvant ADT	0.579	0.093	0.596	0.632	0.637	0.371
	(0.306–1.096)		(0.072–4.949)		(0.237–1.712)	
Adjuvant RT	1.273	0.596	2.892	0.053	0.609	0.563
	(0.521–3.111)		(0.988–8.464)		(0.113–3.275)	
Salvage RT			0.545	0.380	0.832	0.551
			(0.141–2.112)		(0.455–1.523)	
Follow-up duration	1.003	0.144	0.967	<0.001	0.956	<0.001
	(0.999–1.006)		(0.952–0.983)		(0.947–0.964)	
PBT	1.235	0.120	2.362	0.071	1.950	0.006
	(0.946–1.612)		(0.929–6.006)		(1.209–3.145)	
Autologous PBT	1.351	0.095	2.722	0.142	1.040	0.895
	(0.949–1.924)		(0.715–10.361)		(0.580–1.864)	
Allogeneic PBT*	1.341	0.040	4.634	0.004	2.308	0.001
	(1.013–1.775)		(1.618–13.273)		(1.403–3.799)	

* allogeneic with/without autologous PBT

^**+**^ Low-risk: PSA ≤ 10, Gleason score ≤ 6, and clinical stage T1-2a, Intermediate-risk: 10 < PSA < 20, Gleason score 7, or clinical stage T2b, High-risk: PSA ≥ 20, Gleason score ≥ 8, or clinical stage T2c-3a

ADT: androgen deprivation therapy, BMI: body mass index, CSS: cancer-specific survival, EBL: estimated blood loss, ECE: extracapsular extension, Hb: hemoglobin, LN: lymph node, NVB: neurovascular bundle, OS: overall survival, PLND: pelvic lymph node dissection, PSA: prostate-specific antigen, PSM: positive surgical margin, PBT: perioperative blood transfusion, RT: radiotherapy, SVI: seminal vesical invasion.

### Subgroup analysis

From the total patient cohort, 1,663 (62.4%) underwent ORP and 945 (35.4%) underwent RARP (LRP; 59 [2.2%]). In the RARP group, only 47 (4.9%) received PBT. In addition, a significant difference was observed in EBL between the ORP group (834.9ml) and the RARP group (428.2ml) (p < 0.001). Subgroup analysis was performed to adjust for the confounding effects of EBL in patients who underwent ORP with EBL ≥ 1000ml. In the ORP group, 723 (43.5%) patients reported EBL ≥ 1000ml, and among these patients, 160 (22.1%) received PBT (116 [72.5%] patients received allogeneic with/without autologous PBT, and 44 [27.5%] patients received only autologous PBT). The multivariate logistic regression analysis based on EBL (≥ 1000ml vs. < 1000ml) revealed no significant differences between EBL and other pathologic variables (S2 Table). The multivariate Cox regression analysis and Kaplan-Meier survival analysis of this subgroup showed results concordant with the total cohort. Consequently, allogeneic PBT was still significantly associated with BRFS, CSS, and OS; however, autologous PBT was not (data not shown).

## Discussion

Over the past 10 years, the rapid adoption of RARP as the surgical modality of clinically localized prostate cancer has led to lowering of intraoperative EBL and subsequent lower rates of PBT [18, 24, 25]. With this change, the overall PBT rate is decreasing in patients underwent RP. However, ORP is still conducted in a significant portion of RP, and the mean PBT rate has been reported as high as 16.5% (LRP; 4.7%, RARP; 1.8%) [26]. To date, the relationship between PBT and oncologic outcomes has shown conflicting results. Several retrospective studies suggest an association between PBT and mortality in general surgery patients including gastric cancer, hepatocellular carcinoma, and colon cancer [4, 27–29].

In field of bladder cancer, PBT has also been associated with adverse survival outcomes [13, 14, 30]. Abel et al [30]. reported that intraoperative BT, but not postoperative BT, was associated with increased risk of bladder cancer recurrence and mortality. They proposed several mechanisms to explain the association of intraoperative BT with adverse survival outcomes: immunosuppression caused by anesthetics and opioids, release of circulating tumor cells during surgery. However, the association of timing of BT with survival outcomes remains to be researched in prostate cancer. In addition, several studies investigated the impact of blood type on survival outcomes of bladder cancer [31, 32]. They hypothesized that the ABO blood group antigens and the Rhesus factor may influence on survival by various moleculobiologic mechanisms: the ABO antigen expression on the urothelium, the location of ABO blood group on the long arm of chromosome 9 –a commonly altered region in bladder cancer, the encode of ABO gene on specific glycosyl transferases, followed by abnormal glycosylation of cell surface proteins, and then, modulation of epithelial-mesenchymal transition for cancer development and progression. However, these recent studies concluded that the ABO blood group and the Rhesus factor were not associated with survival outcomes in bladder cancer. Tollefson et al. [33] reported that blood type was a significant risk factor for venous thromboembolism after radical prostatectomy with pelvic lymphadenectomy. However, studies investigating the association with survival outcomes in prostate cancer are still lacking.

Allogeneic PBT has been found to be the major cause of TRIM due to transfusion components that mediate immunosuppression, such as allogeneic mononuclear cells, immunosuppressive prostaglandins, soluble biologic response modifiers, and soluble human leukocyte antigen (HLA) Class I peptides [19]. Previous studies of prostate cancer, however, have shown equivalent BRFS for the autologous, allogeneic, and no-PBT groups [7, 8, 16, 22]. In the present study, we found that allogeneic PBT was significantly associated with decreased BRFS, CSS, and OS, while autologous PBT did not show the significant association. These associations persisted after adjusting for potential confounding factors in multivariate analyses, and are also observed in the Kaplan-Meier survival analyses. Consequently, this provides further support for the hypothesis of a TRIM response to allogeneic PBT.

Oefelein et al. [6] reported that the operative EBL, but not the type of transfusion (autologous or allogeneic), was associated with decreased BRFS after RP. They assumed that factors leading to PBT are more significant for outcomes than the immunologic effects of PBT itself. Prior reports showed that PBT is clearly associated with significant EBL during surgery [5, 23] and the current study also showed significant association between PBT and EBL (Table 1). The receipt of PBT might simply be a surrogate marker for more extensive or aggressive disease requiring more aggressive surgical resection, and which is itself an independent predictor of worse oncological outcomes. In order to adjust for EBL as a potential confounding factor, we conducted subgroup analysis in patients who underwent ORP with EBL ≥ 1000ml. Consequently, they showed the same results as those in the total cohort analyses. With this subgroup analyses, we control the confounding factors according to surgical modality and EBL.

Korets et al. [24] found that the date of surgery was a significant predictor for the receipt of PBT within the RARP group. They described that patients who underwent surgery in 2009 or earlier showed a significantly higher risk of receiving PBT compared to the patients underwent surgery in the later years. In current study, we also found that the year of performing the surgery (≥ 2009 vs. < 2009) was a significant predictor of requiring PBT within the RARP group alone, but not within the ORP group (data not shown). We were also able to control this confounding factor with the previously described subgroup analyses conducted in only the ORP group.

The most recent large cohort study conducted in Johns Hopkins Medical Institutions, Chalfin et al. [22] showed that allogeneic but not autologous PBT demonstrated a univariate association with decreased OS. However, the association was no longer significant in the multivariate analyses. This study was comparable to ours; however, the results did not show the concordance seen in other previous studies [7, 8, 15–19]. This discordance might be derived from racial difference in the study population. In contrast with the previous studies, to our knowledge, the current study is the first large cohort study of an Asian (Korean) population. Chhatre et al. [34] showed racial/ethnic differences in elderly patients in Medicare with advanced stage prostate cancer by using SEER-Medicare data. They showed the lower all-cause mortality and prostate cancer-specific mortality in the group of Asian men.

The current study has several limitations. First, our data showed significant clinicopathologic differences between the PBT group and no-PBT group due to the retrospective nonrandomized design (Table 1). As such, possible confounders may not have been completely accounted for even in multivariate analyses. Nevertheless, we did control for these variables in our subgroup analyses models and found that the association of PBT with adverse outcomes was maintained. Second, the administration of PBT was based on the volition of the physicians without institutional standardized criteria. Subsequently, unnecessary PBT might have been received and adversely affected clinical outcomes of patients. With this reason, the rate of PBT in current study was relatively higher than other studies. Third, clinical data describing the use of other blood products such as fresh frozen plasma or platelets was not available. Also, the stratifications according to the timing of BT and the blood type were not accessible. Therefore, we could not evaluate the impact of these variables, which may also be possible confounders and affect clinical outcomes. Fourth, the rate of PLND was relatively lower than other studies. It might be derived from a large portion of RARP (35.4%) in our study population. Prasad et al. [35] reported that the rate of PLND was significantly lower in RARP group than ORP group (17% vs. 83% for LRP/RARP, respectively, p<0.001). In addition, the decision to perform PLND was at the discretion of the surgeon, and then, some had omitted the PLND during RP according to their own clinical judgement. Lastly, follow-up periods were not long enough at 79.4 months in the PBT group and 56.5 months in the no-PBT group (Table 1). The long-term follow-up is essential due to the long-drawn-out clinical course of prostate cancer. Thus, further studies might be necessary to validate our findings.

## Conclusion

In contrast with some previous studies, we found that allogeneic PBT during RP were significantly associated with decreased BRFS, CSS, and OS in both univariate and multivariate analyses. This provides further support for a TRIM hypothesis for allogeneic PBT. While our data are limited to an Asian (Korean) population, the efforts to reduce the use of allogeneic PBT in these patients are warranted. These efforts also include the utilization of intraoperative cell salvage or prepared autologous blood for patients who are expected to receive PBT in preoperative evaluation.

## Supporting Information

S1 Table Clinicopathological parameters of the comparative analysis results according to the receipt of allogeneic and autologous perioperative blood transfusion (PBT). (DOCX)

S2 Table Multivariate logistic regression analyses for evaluating variables associated with EBL (≥1000ml vs. <1000ml) in ORP group. (DOCX)
