# Supplementary material for: Perioperative Blood Transfusion as a Significant Predictor of Biochemical Recurrence and Survival after Radical Prostatectomy in Patients with Prostate Cancer
Source: PLoS One. 2016 May 9;11(5):e0154918. doi: 10.1371/journal.pone.0154918 (PMC4861293; doi:10.1371/journal.pone.0154918)
Supplement: S1 Table — (DOCX) [file pone.0154918.s001.docx]

S1 Table. Clinicopathological parameters of the comparative analysis results according to the receipt of allogeneic and autologous perioperative blood transfusion (PBT).

|  | Allogeneic PBT* | Autologous PBT | p-value |
| --- | --- | --- | --- |
| Patient n (%) = 440 | 350 (79.5 %) | 90 (20.5 %) |  |
| Age, yr, median (SD) | 65.6 (7.6) | 68.6 (6.1) | 0.001 |
| BMI, median (SD) | 24.1 (2.7) | 24.6 (2.7) | 0.154 |
| Preoperative Hb, g/d L, mean (SD) | 13.6 (1.7) | 13.9 (1.2) | 0.213 |
| Neoadjuvant ADT, n (%) |  |  | 0.776 |
| Done | 16 (4.6 %) | 3 (3.3 %) |  |
| Not done | 334 (95.4 %) | 87 (96.7 %) |  |
| Operative type, n (%) |  |  | 0.007 |
| Open | 305 (87.2 %) | 87 (96.7 %) |  |
| Laparoscopic/Robotic | 45 (12.8 %) | 3 (3.3 %) |  |
| PLND, n (%) |  |  | <0.001 |
| Not done | 185 (54.7 %) | 79 (87.8 %) |  |
| Done | 153 (45.3 %) | 11 (12.2 %) |  |
| NVB saving, n(%) |  |  | 0.232 |
| Not done | 173 (80.1 %) | 65 (73.9 %) |  |
| Done | 43 (19.9 %) | 23 (26.1 %) |  |
| Operative time, min, median (IQR) | 201.3 (81.1) | 136.8 (63.1) | <0.001 |
| EBL, ml, median (SD) | 1244.3 (1075.4) | 865.8 (604.1) | <0.001 |
| Preoperative PSA, ng/ml | 12.8 (16.1) | 16.1 (23.0) | 0.212 |
| Pathologic Gleason Score, n (%) |  |  | 0.411 |
| ≤ 8 | 282 (85.2 %) | 71 (81.6 %) |  |
| >8 | 49 (14.8 %) | 16 (18.4 %) |  |
| Pathologic T stage, n (%) |  |  | 0.287 |
| ≤ pT2 | 216 (61.7 %) | 50 (55.6 %) |  |
| ≥pT3 | 134 (38.3 %) | 40 (44.4 %) |  |
| Lymph node status, n (%) |  |  | 0.167 |
| Nx/N0 | 388 (96.6 %) | 84 (93.3 %) |  |
| N1 | 12 (3.4 %) | 6 (6.7 %) |  |
| Total number of removed lymph nodes, mean (SD) | 4.0 (5.7) | 3.0 (6.3) | 0.137 |
| Number of positive lymph nodes,  mean (SD) | 0.06 (0.4) | 0.14 (0.7) | 0.249 |
| ECE, n (%) |  |  | 0.901 |
| Absent | 228 (65.1 %) | 58 (64.4 %) |  |
| Present | 122 (34.9 %) | 32 (35.6 %) |  |
| SVI, n (%) |  |  | 0.570 |
| Absent | 300 (85.7 %) | 75 (83.3 %) |  |
| Present | 50 (14.3 %) | 15 (16.7 %) |  |
| PSM, n (%) |  |  | 0.093 |
| Absent | 228 (65.1 %) | 50 (55.6 %) |  |
| Present | 122 (34.9 %) | 40 (44.4 %) |  |
| Adjuvant ADT, n (%) |  |  | 0.318 |
| Done | 13 (3.7 %) | 1 (1.1 %) |  |
| Not done | 337 (96.3 %) | 89 (98.9 %) |  |
| Adjuvant radiotherapy, n (%) |  |  | 0.208 |
| Done | 10 (3.1 %) | 1 (1.1 %) |  |
| Not done | 340 (96.9 %) | 89 (98.9 %) |  |
| Salvage radiotherapy, n (%) |  |  | <0.001 |
| Done | 68 (19.4 %) | 3 (3.3 %) |  |
| Not done | 282 (80.6 %) | 87 (96.7 %) |  |
| Follow-up, months, median (SD) |  |  |  |
| Biochemical recurrence, n (%) |  |  | <0.001 |
| No | 253 (72.3 %) | 85 (94.4 %) |  |
| Yes | 97 (27.7 %) | 5 (5.6 %) |  |
| CSS result, n (%) |  |  | 0.014 |
| Alive or death from other causes | 333 (95.1 %) | 88 (97.8 %) |  |
| Cancer-specific death | 17 (4.9 %) | 2 (2.2 %) |  |
| OS result, n (%) |  |  | 0.001 |
| Alive | 300 (85.7 %) | 88 (97.8 %) |  |
| All-cause death | 50 (14.3 %) | 2 (2.2 %) |  |

* allogeneic with/without autologous PBT

ADT: androgen deprivation therapy, BMI: body mass index, CSS: cancer-specific survival, EBL: estimated blood loss, ECE: extracapsular extension, Hb: hemoglobin, NVB: neurovascular bundle, OS: overall survival, PLND: pelvic lymph node dissection, PSM: positive surgical margin, PBT: perioperative blood transfusion, SVI: seminal vesical invasion.
