# Supplementary material for: Perioperative Blood Transfusion as a Significant Predictor of Biochemical Recurrence and Survival after Radical Prostatectomy in Patients with Prostate Cancer
Source: PLoS One. 2016 May 9;11(5):e0154918. doi: 10.1371/journal.pone.0154918 (PMC4861293; doi:10.1371/journal.pone.0154918)
Supplement: S2 Table — (DOCX) [file pone.0154918.s002.docx]

S2 Table. Multivariate logistic regression analyses for evaluating variables associated with EBL (≥1000ml vs. <1000ml) in ORP group.

|  | OR | p-value | 95%PI |
| --- | --- | --- | --- |
| PSA<4 | Reference |  |  |
| 4-10 | 1.583 | 0.107 | 0.906-2.767 |
| 10-20 | 1.514 | 0.181 | 0.824-2.780 |
| ≥20 | 1.473 | 0.260 | 0.751-2.887 |
| Pathologic T≥3 | 0.633 | 0.286 | 0.274-1.464 |
| Pathologic GS≥8 | 1.395 | 0.158 | 0.879-2.214 |
| Pathologic LN (+) | 1.956 | 0.054 | 0.915-3.772 |
| ECE (+) | 1.480 | 0.339 | 0.663-3.308 |
| SVI (+) | 0.739 | 0.360 | 0.437-1.251 |
| HGPIN (+) | 0.735 | 0.590 | 0.535-1.010 |
| PSM (+) | 1.384 | 0.052 | 0.923-1.892 |

ECE: extracapsular extension, HGPIN: high-grade prostatic intraepithelial neoplasia, PSM: positive surgical margin, SVI: seminal vesical invasion.
